# Editorial: Advancing antibiotic candidates for eradication of persistent bacterial infections

**DOI:** 10.3389/fphar.2026.1801789

**Published:** 2026-02-23

**Authors:** Laurent A. Bekale, Jessica Amber Jennings, Xilin Zhao

**Affiliations:** 1 Department of Bioengineering, Schools of Engineering and of Medicine, Stanford University, Stanford, CA, United States; 2 Department of Biomedical Engineering, University of Memphis, Memphis, TN, United States; 3 Public Health Research Institute and Department of Microbiology, Biochemistry and Molecular Genetics, New Jersey Medical School, Rutgers Biomedical and Health Sciences, Rutgers University, Newark, NJ, United States

**Keywords:** drug delivery and pharmacokinetics, intracellular infections, metabolomics (^13^C-tracer analysis), *Mycobacterium tuberculosis* persistence, nanomaterial-enabled antimicrobials, phenotypic heterogeneity

Persistent bacterial infections continue to be among the most critical global health challenges. Their stubbornness is often not caused by genetic resistance but by phenotypic persistence, specifically the presence of metabolically inactive bacterial subpopulations that survive otherwise lethal antibiotic exposure. These persister cells, although genetically identical to their susceptible counterparts, enter reversible dormancy states that allow them to tolerate antibiotics and later resuscitate, leading to infection relapse. Recognizing persistence, rather than resistance, as the main cause of chronic and relapsing infections has fundamentally shifted antimicrobial research toward understanding the physiological basis of dormancy and developing therapies that can eliminate non-replicating, metabolically dormant cells.

The Research Topic “Advancing Antibiotic Candidates for Eradication of Persistent Bacterial Infections” integrates mechanistic investigations, pharmacodynamic studies, and conceptual reviews that redefine the approach to the challenge of persistence. Instead of simply compiling antibiotic screening outcomes, the studies collectively highlight how bacterial energy metabolism, intracellular pharmacology, and systems-level targeting must work together to overcome dormancy-associated tolerance.

Sun et al. provide a compelling metabolic perspective, applying ^13^C-tracer analysis and isotope-assisted LC–MS/GC–MS to dissect carbon flux in *Escherichia coli* persister populations (Sun et al.). Their work demonstrates that metabolic dormancy is not an absolute shutdown, but a gradient state modulated by nutrient source and intracellular ATP barriers. Glucose-fed persisters maintain residual flux through glycolytic intermediates, while acetate-fed cells exhibit deeper dormancy linked to energy depletion and redox stress. This study refines the long-held view of persisters as metabolically inert, revealing that selective metabolic awakening may render them vulnerable to antibiotics. It highlights energy restoration pathways as actionable intervention points for reactivation-based killing strategies, a concept gaining traction across persistent infection models.

Huguet et al. examine a crucial aspect of persistence and survival within host cells. Using *Rhodococcus equi*, an intracellular pathogen that affects both animals and immunocompromised humans, the authors compare the bactericidal effects of macrolides and their combinations (Huguet et al.). Notably, they evaluate antibiotic effectiveness at concentrations relevant to physiological conditions, such as in epithelial lining fluid and macrophage compartments. Clarithromycin proved to be the most effective monotherapy, and combinations of clarithromycin with doxycycline showed additive intracellular killing. This pharmacodynamic approach highlights how antibiotic efficacy is greatly affected by subcellular drug distribution and the infection microenvironment. The study demonstrates how modeling the infection environment can connect *in vitro* susceptibility to *in vivo* effectiveness, marking an important step toward rational combination therapy.

Focusing on translational application and efficiency in *in vivo* testing, Tian et al. introduces a novel autoluminescence-based inhalation administration model for assessing drug activity against *Mycobacterium tuberculosis* (Tian et al.). Traditional models for this major persistent pathogen were often intricate, time-consuming, and lacked reproducibility due to complex procedures like anesthesia and invasive sampling. This new non-invasive approach utilizes autoluminescent *M. tuberculosis* in live mice, directly quantifying relative light units (RLUs) as a surrogate marker for colony-forming units (CFUs) to assess drug efficacy. The model dramatically shortens the evaluation time from months to just 16–17 days, offering a cost-effective, high-throughput method for the objective evaluation of inhalable drugs like rifampicin, isoniazid, and ethambutol. This innovation represents a critical advancement in overcoming the pharmacokinetic limitations of conventional anti-TB therapies by facilitating the rapid and accurate *in vivo* screening of delivery methods optimized for high local concentration in the lungs.

Hashemi et al. integrate these mechanistic and pharmacologic insights into a comprehensive framework for persister eradication (Hashemi et al.). Their review categorizes therapeutic approaches into four mechanistic classes: (i) direct killing via membrane disruption; (ii) inhibition of persister formation; (iii) synergistic combination therapy; and (iv) exploitation of dormancy depth. Emerging technologies, such as nanomaterial adjuvants (e.g., gold nanoclusters) and proteolysis-targeting activators (e.g., ADEP4 and ClpP modulators), exemplify how chemical biology and nanotechnology are broadening the anti-persister toolkit. This synthesis underscores a paradigm shift from single-agent antibiotic development toward mechanism-driven, multi-modal therapies that incorporate bioenergetic disruption, host environment modeling, and drug-delivery innovation.

Collectively, the contributions to this Research Topic converge on a unifying principle: bacterial persistence represents a dynamic, targetable physiological state, not a fixed obstacle. Persisters maintain minimal yet sufficient metabolic flux to sustain survival, and this metabolic flexibility can be leveraged for therapeutic intervention. Intracellular infection models show that antibiotic effectiveness is profoundly shaped by environmental context, including oxygen tension, nutrient availability, and host cell metabolism. Combining these aspects yields a more predictive understanding of drug efficacy against dormant bacteria. The central implication is clear: overcoming persistence requires a systemic approach that integrates microbiology, pharmacology, and materials science. The convergence of isotope-resolved metabolomics, physiologically relevant pharmacokinetic models, and nanoscale drug design marks a transition from empirical screening toward a rational, systems pharmacology of persistence.

While research on this Research Topic highlights key aspects of bacterial persistence, it also exposes the complexity that continues to hinder therapeutic progress. Eradicating persister cells will require a fundamental shift from descriptive microbiology to predictive systems pharmacology, where metabolism, drug dynamics, and host interactions are modeled as interconnected variables rather than isolated phenomena. To fully understand bacterial persistence as a pharmacologically targetable state, the research roadmap must explore four complementary avenues.

1. Identifying the key energy bottlenecks that support survival under antibiotic stress, providing logical entry points for either metabolic reactivation or collapse.
2. Evaluating combination strategies within host tissue microenvironments to uncover synergistic vulnerabilities that are not evident in standard *in vitro* assays.
3. Developing persistence models that integrate host–pathogen interactions, intracellular niches, and immune modulation for translating discoveries into clinically viable drug candidates.
4. Using omics-guided profiling and real-time imaging to refine dosing regimens, monitor drug penetration and bacterial energy states, and identify quantitative biomarkers of eradication.

Progressing along these four axes will turn persistence from a mysterious survival phenomenon into a predictable, preventable, and ultimately curable state of bacterial physiology.

